# Synthesis methods used to combine observational studies and randomised trials in published meta-analyses

**DOI:** 10.1186/s13643-024-02464-w

**Published:** 2024-02-21

**Authors:** Cherifa Cheurfa, Sofia Tsokani, Katerina-Maria Kontouli, Isabelle Boutron, Anna Chaimani

**Affiliations:** 1 Université Paris Cité and Université Sorbonne Paris Nord, Inserm, INRAe, Centre for Research in Epidemiology and Statistics (CRESS), Hôpital Hôtel Dieu, 1 Place du Parvis Notre-Dame, 75004 Paris, France; 2 Department of Anesthesiology and Critical Care, AP-HP, Cochin Hospital, 75004 Paris, France; 3 Department of Primary Education, School of Education, University of Ioannina, Ioannina, Greece; 4 Centre d’Épidémiologie Clinique, AP-HP, Hôpital Hôtel-Dieu, 75004 Paris, France; 5 Cochrane France, Paris, France

**Keywords:** Data synthesis, Non-randomised studies, Comparative effectiveness, heterogeneous designs

## Abstract

**Background:**

This study examined the synthesis methods used in meta-analyses pooling data from observational studies (OSs) and randomised controlled trials (RCTs) from various medical disciplines.

**Methods:**

We searched Medline via PubMed to identify reports of systematic reviews of interventions, including and pooling data from RCTs and OSs published in 110 high-impact factor general and specialised journals between 2015 and 2019. Screening and data extraction were performed in duplicate. To describe the synthesis methods used in the meta-analyses, we considered the first meta-analysis presented in each article.

**Results:**

Overall, 132 reports were identified with a median number of included studies of 14 [9–26]. The median number of OSs was 6.5 [3–12] and that of RCTs was 3 [1–6]. The effect estimates recorded from OSs (i.e., adjusted or unadjusted) were not specified in 82% (*n* = 108) of the meta-analyses. An inverse-variance common-effect model was used in 2% (*n* = 3) of the meta-analyses, a random-effects model was used in 55% (*n* = 73), and both models were used in 40% (*n* = 53). A Poisson regression model was used in 1 meta-analysis, and 2 meta-analyses did not report the model they used. The mean total weight of OSs in the studied meta-analyses was 57.3% (standard deviation, ± 30.3%). Only 44 (33%) meta-analyses reported results stratified by study design. Of them, the results between OSs and RCTs had a consistent direction of effect in 70% (*n* = 31). Study design was explored as a potential source of heterogeneity in 79% of the meta-analyses, and confounding factors were investigated in only 10% (*n* = 13). Publication bias was assessed in 70% (*n* = 92) of the meta-analyses. Tau-square was reported in 32 meta-analyses with a median of 0.07 [0–0.30].

**Conclusion:**

The inclusion of OSs in a meta-analysis on interventions could provide useful information. However, considerations of several methodological and conceptual aspects of OSs, that are required to avoid misleading findings, were often absent or insufficiently reported in our sample.

**Supplementary Information:**

The online version contains supplementary material available at 10.1186/s13643-024-02464-w.

## Background

The incorporation of non-randomised evidence in meta-analyses has attracted a lot of interest [1–3]. The results of observational studies (OSs) may have high external validity and generalizability [4–6]. However, there are concerns regarding the risk of bias in OSs, particularly selection and confounding bias, and the large heterogeneity in their study designs [7–11].

Several meta-epidemiological studies have investigated the similarity between the results of randomised controlled trials (RCTs) and OSs for the same research questions [12–14]. Bun et al. included 102 meta-analyses of OSs and RCTs and showed that the average treatment effects did not differ substantially between the two study designs [12]. Similar findings were obtained by Golder et al. [13]. Furthermore, Beyerbach et al. suggested that not only is evidence from RCTs and OSs usually in agreement but that the inclusion of observational data is crucial to obtain a global view of the available evidence in the field of nutrition [14, 15].

Despite the above findings, these empirical investigations have focused only on the similarity of the numerical summaries between the two types of studies. This approach ignores the inevitable clinical and methodological heterogeneity between RCTs and OSs, which is not always reflected in the observed treatment effects of individual studies [16]. In addition, more and less heterogeneous datasets may provide similar summary results as the weighted average can remain unchanged when adding equally extreme effects of similar precision to its left and right. Focusing only on the overall effect may cause us to overlook important discrepancies between or within subgroups [2, 17, 18]. In such cases, the risk of establishing misleading conclusions is high, and the interpretation of summary results from highly heterogeneous sets of studies should explicitly account for the inherently high uncertainty they are accompanied with [19–21]. Further, the interpretation of OS findings depends on the confounding factors that have been adjusted for. Hence, the use of different adjustment factors across OSs may challenge the comparability of their findings as well as with RCTs [22, 23].

We have previously explored the approaches that review authors used at the systematic review stage (data extraction, risk of bias assessment, etc.) to reassure that the specific challenges and potential biases introduced by the inclusion of observational studies will be adequately accommodated in their conclusions [24]. We found that published systematic reviews including RCTs and OSs were often lacking proper reporting and methodology for OSs; for instance, few reviews only reported registration of a protocol and adjusted estimates were rarely extracted. In this study, we investigated the synthesis methods that have been used to combine the findings of RCTs and OSs and to explore the potential discrepancies in their results. We included studies published in general and specialised high impact factor journals and focused on their statistical approaches for data synthesis, evaluation and exploration of statistical and clinical heterogeneity, handling of confounders and potential reporting biases in the analysis, and interpretation of results.

## Methods

The search strategy and selection process for eligible systematic reviews have been previously described in detail [24]. Briefly, we searched Medline via PubMed to identify systematic reviews that included RCTs and OSs evaluating the effect of healthcare interventions, published between January 2015 and December 2019 in general and internal medicine or public health journals with an impact factor ≥ 2.5 or in the top five specialty medical journals. The full search strategy is presented in Additional file 1. We included systematic reviews with at least one meta-analysis pooling the results of RCTs and OSs and we considered the first meta-analysis presented in each article. Systematic reviews with meta-analyses that included fewer than five studies in total were excluded. Two reviewers (CC and CL) performed the selection of the meta-analyses.

### Data extraction

Data were systematically extracted from each article using a preestablished data extraction form (Additional file 2).

#### General characteristics

We recorded the journal type (specialty or general medical journal), type of treatment evaluated, review eligibility criteria, type of OSs included, any design-specific analyses for RCTs and different types of OSs, and median number of studies included overall and under each study type. We assessed whether and how the risk of bias in the primary studies was evaluated and whether any levels of risk of bias were excluded from the systematic review.

#### Synthesis methods

We recorded their eligibility criteria; we also checked whether criteria, such as study type, sample size, or risk of bias thresholds, were applied at the synthesis level in addition to the inclusion criteria at the systematic review level. We determined the outcome data that were extracted for the analyses of OSs (i.e., adjusted or unadjusted estimates). We further extracted the synthesis model used and determined whether and how confounding factors had been considered before or during the synthesis (e.g. restricting the meta-analysis to adjusted estimates for all important confounders) [25].

In addition, we recorded whether and how review authors assessed and explored heterogeneity and whether the study design or other study characteristics were considered as potential sources of heterogeneity. We determined whether the review authors assessed reporting bias (graphically, statistically, or both) and, if not, whether they provided a justification (e.g., insufficient number of studies).

We extracted the total number of RCTs and OSs synthesised, outcomes for which the two study designs were combined, and specific randomised and non-randomised designs that were included or excluded.

We determined the total weight of each design in the estimation of the summary effect and whether a subgroup analysis by study design was performed. We also recorded how the confounding factors and heterogeneity were accounted for in the interpretation of the results and final conclusions.

We assessed the consistency between the pooled results of OSs and RCTs by visual inspection of the summary effects and their confidence intervals. Specifically, we monitored whether the overall effects were in the same direction and whether they were significant. We extracted the test for subgroup differences when this was reported.

### Statistical analysis

We summarised our findings using descriptive statistics. Categorical variables are presented as frequencies and percentages and continuous variables as medians with interquartile ranges (IQR). All analyses were performed using the R software [26] and some of the figures were created using the Excel Stat software [27].

## Results

**Fig. 1:**
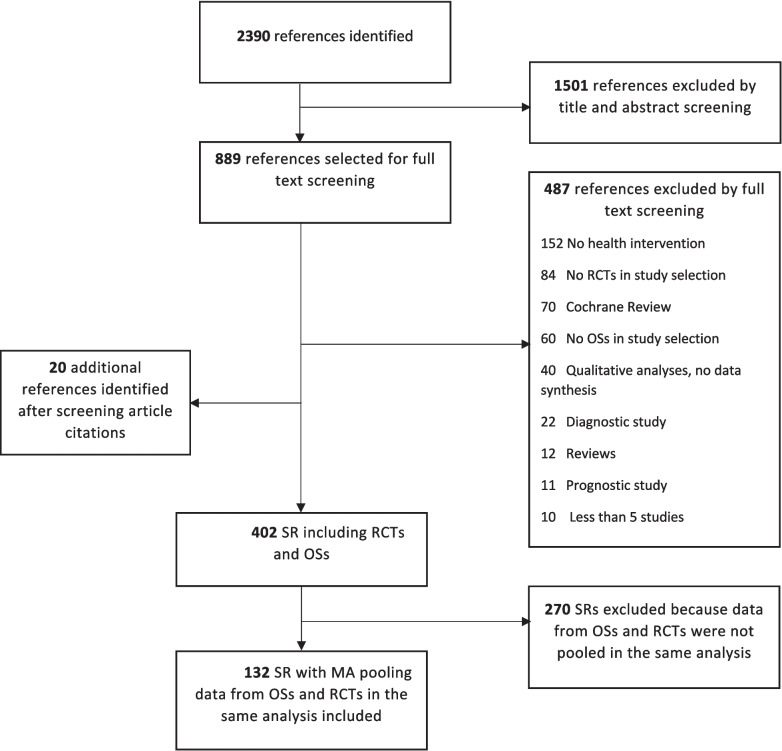
Flow chart of study selection

We identified 402 systematic reviews that included both RCTs and OSs. Of them, 132 (33%) reviews that pooled data from RCTs and OSs were included in this study (Additional file 3). The flowchart of the selection process is shown in Fig. 1. The PRISMA checklist has been attached as an additional file (Additional file 4).

### General characteristics of the reviews (Table 1)

**Table 1:** Characteristics of the included systematic review reports

Characteristic	Studies
	*N* = 132
	n (%)
Journal of publication	
Specialty	119 (90)
General	33 (10)
Country	
Europe	44 (33)
North America	32 (24)
Middle/South America	1 (1)
Australia	7 (5)
Asia	48 (37)
Africa	0 (0)
Type of intervention	
Pharmacological	51 (39)
Non-pharmacological	81 (61)
Primary studies included (median [IQR])	14 [9.0–26.0]
Randomised controlled trials included (median [IQR])	5.0 [2.0–8.0]
OSs included (median [IQR])	9. 0 [5.0–18.0]
Specific OS designs included	
Prospective cohort studies	17 (13)
Retrospective cohort studies	13 (10)
Case control studies	9 (7)
Were the different types of OSs analysed differently?	
Yes	8 (6)
No	120 (91)
Unclear	4 (3)
Was an evaluation of the risk of bias for primary studies provided?	
yes, with different tools for OSs and RCTs	79 (65)
yes, with same tool for OSs and RCTs	33 (27)
yes, for OSs only	8 (6)
yes, for RCTs only	1 (1)
No	11 (8)

*MA* Meta-analysis, *OSs* Observational studies, *RCTs* Randomised controlled trials, *RoB* Risk of bias

The reviews were mainly published in specialty journals, and 61% of them assessed non-pharmacologic interventions.

The median [IQR] number of studies included in the reviews was 14 [9-26]: 5 [2-8] RCTs and 9 [5-18] OSs. Of the systematic reviews, 13% (*n* = 17) exclusively included prospective cohort studies, 10% (*n* = 13) exclusively included retrospective cohort studies, and 7% (*n* = 9) exclusively included case–control studies.

Overall, 92% (*n* = 121) of the reviews assessed the risk of bias of the primary studies. Tools specific to the study design were used in 65% (79/121) of the reviews, whereas 27% (*n* = 33/121) used the same tool for both study designs; 6% (*n* = 8/121) assessed the risk of bias of OSs alone, and 1% (*n* = 1/121) assessed the risk of bias of RCTs alone.

### Synthesis methods (Table 2)

**Table 2:** Characteristics of the synthesis methods

Characteristic	Studies
	n (%)
Primary studies included (median [IQR])	10 [7–17]
Number of RCTs included (median [IQR])	3 [1–6]
Number of OSs included (median [IQR])	6.5 [3–12]
Type of outcome analysed	
Primary analysis	113 (86)
Secondary analysis	12 (9)
Efficacy outcome	84 (64)
Safety outcome	28 (21)
Type of data pooled	
Adjusted	7 (5)
Not adjusted	5 (4)
Both adjusted and non-adjusted	7 (5)
Other	5 (4)
Not reported	108 (82)
Synthesis model	
Random effects model	73 (55)
Common effect model	3 (2)
Both models	53 (40)
Other	1 (1)
Not reported	2 (2)
Types of effects reported	
Effect estimates for each primary study	121 (92)
Meta-analysis summary effect	77 (58)
Effects stratified by study design	44 (34)
Statistical heterogeneity evaluation	*N* = *132*
I2	123 (93)
Q test	25 (19)
Tau	32 (24)
Statistical heterogeneity exploration	*N* = *105*
Sensitivity analyses	71 (68)
Subgroup analyses	75 (70)
Meta-regression	*23 (22)*
Source of heterogeneity studied by the meta-analyses’ authors	*N* = *105*^a^
Study design	283 (79)
Risk of bias of the primary studies	47 (45)
Study population characteristics	37 (35)
Other confounding factors	11 (10)
Outcome assessment	24 (23)
Publication bias and small study effect evaluation	
Graphical evaluation	85 (64)
Statistical evaluation	66 (50)
No evaluation	40 (30)

^a^Number of MAs exploring sources of heterogeneity. *MA* Meta-analysis, *OSs* Observational studies, *RCTs* Randomised controlled trial

#### General characteristics

The included meta-analyses selected were primary analyses in 86% (*n* = 113) of the reviews. It pertained to an efficacy outcome in 64% and to a safety outcome in 21% (*n* = 28). The median number of studies included was 14 [9-26], with a median of 6.5 [3-12] OSs and 3 [1-6] RCTs.

#### Methodological characteristics

No additional restrictions on OS design, sample size, or any other parameters were imposed for inclusion in the meta-analysis. Nevertheless, high risk of bias OSs were excluded post-hoc in six meta-analyses.

Overall, 82% (*n* = 108) of the meta-analyses did not specify the effect estimates recorded from OSs (i.e., adjusted or unadjusted). Of the meta-analyses that reported the type of effect estimates (*n* = 24), 9% (*n* = 12) used adjusted estimates or estimates obtained using propensity scores and other matching techniques (stratified or weighted); 4% (*n* = 5) used unadjusted estimates only; and 5% (*n* = 7) used both adjusted and unadjusted estimates.

#### Statistical characteristics

Meta-analyses were performed using an inverse-variance common-effect model in 2% (*n* = 3), a random-effects model in 55% (*n* = 73), both models in 40% (*n* = 53), and a Poisson regression model in one meta-analysis. Two meta-analyses did not report the model used. The choice of a common-effect model was justified by the small number of included studies (*n* = 2) and the absence of important heterogeneity (*n* = 1).

A forest plot was presented in 98% of the selected meta-analyses (*n* = 129). Overall, 98% (*n* = 129) provided the effect estimates and the weight of each primary study. The mean total weight of OSs was 57.3% (standard deviation, ± 30.3%). Only 44 (33%) meta-analyses reported results stratified by study design. Among them, the results of OSs and RCTs were consistent in direction in 70% (*n* = 31) of the meta-analyses.

#### Heterogeneity (Table 2)

Statistical heterogeneity was assessed based on the I^2^ in 93% (*n* = 123) of the meta-analyses, on Q-test in 19% (*n* = 25), and on the between-study variance (tau-square) in 24% (*n* = 32). The I^2^ percentage was > 50% in 49% (*n* = 62) of the meta-analyses. Regarding the Q-test, 16 (12%) reviews reported a *p*-value < 0.05. The median of tau-square reported in 32 meta-analyses was 0.07 [0–0.30].

Statistical heterogeneity was explored in 80% (*n* = 105) of the above 123 meta-analyses that assessed heterogeneity. Out of these 105 meta-analyses, 68% (*n* = 71) explored heterogeneity using sensitivity analyses, 70% (*n* = 75) using subgroup analyses, and 22% (*n* = 23) using meta-regression. Study design was explored as a potential source of heterogeneity in 79% (*n* = 83) of the 105 meta-analyses. Of the latter 83 meta-analyses, 64% (*n* = 56) also considered the different types of OSs, such as prospective or retrospective and cohort or case control studies. The additional sources of heterogeneity explored included study population characteristics in 35% (*n* = 37) of the meta-analyses, risk of bias of primary studies in 45% (*n* = 47), outcome assessment in 23% (*n* = 24), and other characteristics that may act as confounders in 10% (*n* = 11) (Table 3). Out of the 75 meta-analyses that performed subgroup analysis, 24 reported the test for subgroup differences. In two of them the test was statistically significant and in one of these two the effects were in opposite directions.

**Table 3:** Limitations related to study design reported by authors

Characteristic	Studies
	*N* = 132
	n (%)
Limitations related to study design	
Reported	93 (70)
High RoB	61 (48)
Heterogeneity	70 (56)
Population	50 (40)
Intervention	15 (12)
Outcome	11 (9)
Publication bias	6 (5)
Small study effects	5 (4)
Not reported	39 (30)
Concerns regarding the synthesis of OSs with RCTs	
Reported	32 (24)
Risk of bias	13 (10)
Inclusion of OSs and their interpretation	9 (7)
Small number of available RCTs	7 (5)
Risk of confounding	4 (3)
Extraction of effect estimate	2 (2)
Just caution on interpretation	5 (4)
Not reported	100 (81)

MA Meta-analysis, *OSs* Observational studies, *RCTs* Randomised controlled trial, *RoB* Risk of bias

#### Confounding factors (Fig. 2)

**Fig. 2:**
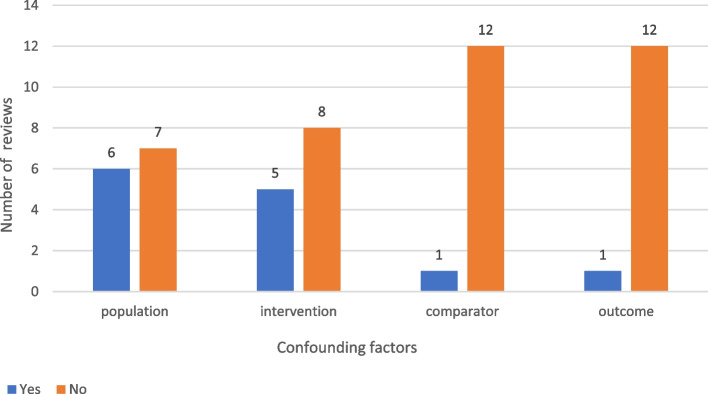
Confounding factors explored in the meta-analyses

In 10% (*n* = 13) of the meta-analyses, the authors reported that confounding factors were not sufficiently controlled for in OSs. These were factors related to the population (5%; *n* = 6) (e.g., age, sex, or comorbidities), the intervention itself (4%; *n* = 5) (e.g., way of administration or type of injection), the comparator in (1%; *n* = 1), and the outcomes (1%, *n* = 1) (e.g., symptoms, severity, or stage of disease). The impact of these factors was investigated at the meta-analysis level through sensitivity analyses (*n* = 7), meta-regression (*n* = 3), stratification (*n* = 2), and data transformation (*n* = 1) [28].

#### Publication bias and small study effect

The risk of small-study effects and publication bias was assessed using graphical methods (i.e., funnel plots) in 64% (*n* = 85) of the meta-analyses and statistical methods (e.g., Egger’s test) in 50% (*n* = 66). Out of the 85 that used funnel plots, 42 (49%) explored whether small-study effects are due to publication bias through contour-enhanced funnel plots. Regarding the meta-analyses that did not assess publication bias (*n* = 40), 90% (*n* = 36) did not justify this choice.

#### Limitations reported by authors (Table 3)

Of the total 132 meta-analyses, only 24% (*n* = 32) expressed some concerns regarding the validity of their results. These concerns included the presence of high risk of bias studies (*n* = 13, 10%), the inclusion of OSs and their interpretation (*n* = 9, 7%), the small number of available RCTs (*n* = 7, 5%), the effect estimates extracted (adjusted or not) (*n* = 2, 2%), the risk of confounding (*n* = 4, 3%). Only 21% (*n* = 28) of the meta-analyses used the GRADE system to critically appraise the findings.

## Discussion

In this study, we investigated the methods used by published meta-analyses involving both OSs and RCTs. We included 402 meta-analyses that synthesised a total of 2791 RCTs and 6820 OSs and were published between 2015 and 2019. To our knowledge, this is the largest sample of meta-analyses considering the combination of the two study designs, with 132 (33%) of them having undertaken quantitative synthesis for at least one outcome. Overall, we identified several methodological deficiencies with respect to the inclusion of OSs, suggesting that review authors may not always be aware of the challenges imposed by the synthesis of non-randomised studies. In particular, the risk of confounding in OSs was rarely considered a threat to the validity of the results, with the majority of reviews either extracting unadjusted estimates or not reporting the type of OS estimates they synthesised. Despite the large heterogeneity anticipated in the meta-analyses of OSs, few of the included reviews explored the observed heterogeneity or considered study design as a contributing factor to between-study variability. However, two-thirds of the included systematic reviews did not pool OSs and RCTs; this suggests that implicitly the review authors often consider such a synthesis inappropriate or challenging.

Several studies have shown that meta-analyses combining different designs tend to be more heterogeneous than those that include only RCTs. Further, the nature of the estimates used from OSs (adjusted or unadjusted estimates) can substantially affect the results of the meta-analysis [12]. A meta-epidemiological study including 19 meta-analyses on side effect outcomes suggested that although on average there was no significant difference between the results of RCTs and OSs, restricting the analysis to only RCTs or OSs may yield different conclusions than those obtained with their combination [13].

Our results have important implications for evidence synthesis. The inclusion of OSs in meta-analyses could provide valuable information, but if it is not performed properly, the conclusions may be misleading [3]. In meta-analyses involving OSs, it is essential to predefine the most important confounding factors and seek OS estimates adjusted for at least these factors [29, 30]. In addition, review authors should bear in mind that important discrepancies do not only exist between OSs and RCTs but also among the different types of OSs (studies with diverse populations, follow-up durations, and risk of bias, including publication bias). However, such differences may not always be reflected in the observed relative effects, making the evaluation of clinical and methodological heterogeneity crucial. The Cochrane Handbook also recommends excluding highly biased OSs from meta-analyses [31].

Our study has some limitations. First, our analysis was based on published reports, and some of the identified issues might be due to poor reporting rather than poor methodology. Furthermore, although our sample was large and covered a variety of medical specialties, it remains non-exhaustive. In addition, our last search was in June 2019 and therefore our results might not capture any improvements due to the publication of the new Cochrane Handbook that was published in 2019 [31]. However, empirical evidence has suggested that published guidelines may need several years to start having an impact on research practice [32]. Finally, we did not quantitatively estimate the discrepancy in the results between OSs and RCTs, as has been estimated by previous meta-epidemiological studies.

## Conclusion

In conclusion, OSs may be equally or even more relevant to certain research questions, and meta-analysts should consider their inclusion. However, our study highlights the need to develop concrete guidance on methods for synthesising different study designs within systematic reviews and meta-analyses.

### Supplementary Information

**Additional file 1.** Search strategy.

**Additional file 2.** Data extraction form.

**Additional file 3.** References of 132 studies included.

**Additional file 4.** PRISMA Checklist for research.

## Data Availability

The datasets used and analysed in the current study are available at the Open Science Framework platform (10.17605/OSF.IO/7JEP3).

## Abbreviations

OS: Observational study
RCT: Randomised controlled trial
