# Supplementary material for: Synthesis methods used to combine observational studies and randomised trials in published meta-analyses
Source: Syst Rev. 2024 Feb 21;13:70. doi: 10.1186/s13643-024-02464-w (PMC10880204; doi:10.1186/s13643-024-02464-w)
Supplement: Supplementary file 1 — Additional file 1. Search strategy. [file 13643_2024_2464_MOESM1_ESM.docx]

**Additional file 1.** Search Strategy

| #1 | (meta-analys*[Title] OR (systematic review[Title] AND (synthes* OR pool* OR combin*))) AND (observational OR cohort OR case-control OR proensity score* OR cross-sectional OR single-arm OR non-randomi*) NOT (diagnostic[Title] OR protocol[Title] OR overview[Title] OR meta-epidemiol*[Title] | 49116 |
| --- | --- | --- |
| #2 | ("N Engl J Med"[Journal] OR “JAMA”[Journal] OR “Lancet”[Journal] OR "Ann Intern Med"[Journal] OR "Plos Med"[Journal] OR "Int J Epidemiol"[Journal] OR “BMJ”[Journal] OR "BMC Med"[Journal] OR "Health Technol Assess"[Journal] OR "Plos One"[Journal] OR "Cochrane Database Syst Rev"[Journal] OR "Am J Epidemiol"[Journal] OR "J Clin Epidemiol"[Journal] OR "Nat Med"[Journal] OR "Mayo Clin Proc"[Journal] OR "CMAJ"[Journal] OR "J Intern Med"[Journal] OR "J Clin Med"[Journal] OR "Am J Med"[Journal] OR "Med J Aust"[Journal] OR "J Gen Intern Med"[Journal] OR "Epidemiol rev"[Journal] OR "Eur J Epidemiol"[Journal] OR "Epidemiology"[Journal] OR "Am J Public Health"[Journal] OR "J Glob Health"[Journal] OR "J Epidemiol Community Health"[Journal] OR "Clin Epidemiol"[Journal] OR "Cancer epidemiol"[Journal] OR "Ann Epidemiol"[Journal] OR "Eur J Public Health"[Journal] OR "J Med Screen"[Journal] OR "Int J Public Health"[Journal] OR "J Epidemiol"[Journal] OR " BMC Public health "[Journal] OR "Eur Heart J"[Journal] OR "J Am Coll Cardiol"[Journal] OR "JACC Cardiovasc Interv"[Journal] OR "Eur J Heart Fail"[Journal] OR "JAMA Cardiol"[Journal] OR "Neurosci Biobehav Rev"[Journal] OR "Brain Stimul"[Journal] OR "Neuropsychopharmacology"[Journal] OR "Pain"[Journal] OR "Eur J Neurol"[Journal] OR "Ann Surg"[Journal] OR "Br J Surg"[Journal] OR "J Thorac Cardiovasc Surg"[Journal] OR "Plast Reconstr Surg"[Journal] OR "J Neurosurg"[Journal] OR "Lancet Psychiatry"[Journal] OR "JAMA Psychiatry"[Journal] OR "Am J Psychiatry"[Journal] OR "J Alzheimers Dis"[Journal] OR "Eur Child Adolesc Psychiatry"[Journal] OR "Lancet Respir Med"[Journal] OR "Eur Respir J"[Journal] OR "Chest"[Journal] OR "Lung Cancer"[Journal] OR "Respirology"[Journal] OR "Lancet Infect Dis"[Journal] OR "Lancet HIV"[Journal] OR "Clin Infect Dis"[Journal] OR "Clin Microbiol Infect"[Journal] OR "J Infect Dis"[Journal] OR "Lancet Oncol"[Journal] OR "J Clin Oncol"[Journal] OR "JAMA Oncol"[Journal] OR "Ann Oncol"[Journal] OR "Neuro Oncol"[Journal] OR "Ann Rheum Dis"[Journal] OR "Osteoarthritis Cartilage"[Journal] OR "J Rheumatol"[Journal] OR "Arthritis Care Res (Hoboken)"[Journal] OR "Arthritis Res Ther"[Journal] OR "JAMA Intern Med"[Journal] OR "Lancet Haematol"[Journal] OR "Haematologica"[Journal] OR "Arterioscler Thromb Vasc Biol"[Journal] OR "Am J Hematol"[Journal] OR "Thromb Haemost"[Journal] OR "Anesthesiology"[Journal] OR "Br J Anaesth"[Journal] OR "Eur J Anaesthesiol"[Journal] OR "J Clin Anesth"[Journal] OR "Anesth Analg"[Journal] OR "Intensive Care Med"[Journal] OR "Crit Care Med"[Journal] OR "Resuscitation"[Journal] OR "J Neurotrauma"[Journal] OR "J Trauma Acute Care Surg"[Journal] OR "Gastroenterology"[Journal] OR "Gut"[Journal] OR "Lancet Gastroenterol Hepatol"[Journal] OR "Am J Gastroenterol"[Journal] OR "Clin Gastroenterol Hepatol"[Journal] OR "Radiology"[Journal] OR "J Nucl Med"[Journal] OR "Ultrasound Obstet Gynecol"[Journal] OR "Radiother Oncol"[Journal] OR "Eur Radiol"[Journal] OR "JAMA Pediatr"[Journal] OR "J Am Acad Child Adolesc Psychiatry"[Journal] OR "Pediatr Allergy Immunol"[Journal] OR "J Pediatr"[Journal] OR "Dev Med Child Neurol"[Journal] OR "Eur Urol"[Journal] OR "Am J Kidney Dis"[Journal] OR "Clin J Am Soc Nephrol"[Journal] OR "J Urol"[Journal] OR "Prostate Cancer Prostatic Dis"[Journal]) | 371529 |
| #3 | #1 + #2 | 2390 |
