# Supplementary material for: Synthesis methods used to combine observational studies and randomised trials in published meta-analyses
Source: Syst Rev. 2024 Feb 21;13:70. doi: 10.1186/s13643-024-02464-w (PMC10880204; doi:10.1186/s13643-024-02464-w)
Supplement: Supplementary file 2 — Additional file 2. Data extraction form. [file 13643_2024_2464_MOESM2_ESM.docx]

**Additional file 2**. Data Extraction form

***Methodological characteristics of Systematic Review*:**

*1-General characteristics*

- Intervention type: 🞎 Pharmacological Interventions

🞎 Non-Pharmacological Interventions

🞎 Other

*2-Methodological characteristics*

- Are the eligibility criteria the same for OS and RCTs, outside study design? 🞎 Yes

🞎 No

- Eligibility criteria(inclusion) specific to OS:

🞎 Sample size threshold for inclusion: ⭘ Yes, at the SR level

⭘ Yes, at the MA level

⭘ No

⭘ If Yes, which?

🞎 Specific design excluded for OS: ⭘ Yes, at the SR level

⭘ Yes, at the MA level

⭘ No

⭘ If Yes, which: Retrospective studies

Cross sectional studies

Electronic databases

Other

⭘ If Yes, why: ROB

Other

🞎 Specific design included for OS: ⭘ Yes, at the SR level

⭘ Yes, at the MA level

⭘ No

⭘ If Yes, which: Cohort studies

Cross sectional studies

Interrupted time series (ITS)

Instrumental variable estimation (IVE)

Non-randomized controlled trial(N-RCT)

Case series Case control

Other

🞎 risk of bias threshold for inclusion: ⭘ Yes, at the SR level

⭘ Yes, at the MA level

⭘ No

⭘ If Yes, which: Only low

Medium or low

🞎 Availability of outcome: ⭘ Yes, at the SR level

⭘ Yes, at the MA level

⭘ No

🞎 Type of analysis of included studies: ⭘ Yes, at the SR level

⭘ Yes, at the MA level

⭘ No

⭘ If Yes, which?

-inverse probability weighting

-analysis of propensity scores

-analysis considering prespecified

cofounding factors

- analysis considering the presence of co-interventions

-other

🞎 Other

- Eligibility criteria(inclusion) specific to RCTs:

🞎 Sample size threshold for inclusion: ⭘ Yes

⭘ No

⭘ If Yes, which?

🞎 Specific design excluded for RCTs: ⭘ Yes

⭘ No

⭘ If Yes, which:

Cluster-randomized trials

Cross over trials

Other

⭘ If Yes, why: ROB

Other

🞎 risk of bias threshold for inclusion: ⭘ Yes

⭘ No

⭘ If Yes, which: Only low

Medium or lower

🞎 Availability of outcome: ⭘ Yes

⭘ No

🞎 Type of analysis of included studies: ⭘ Yes

⭘ No

⭘ If Yes, which?

🞎 Other

- Did authors assess the ROB of primary studies?

🞎 Yes only for RCTs

🞎 Yes for both designs with the same tool

🞎 Yes for both designs with different tools

🞎 No

- Did authors use the same tool for OS whatever the design? 🞎 Yes

🞎 No

- Methods used for assessing risk of bias of individual studies:

***🡪 Observational studies***

🞎 Tool’s for assessing OS:

⭘ Cochrane Risk of bias tool

⭘ Newcastle-Ottawa Scale

⭘ ROBINS-I tool

⭘ STROBE

⭘ Effective Practice and Organization of Care group for interrupted time series studies (ITS)

⭘ No standardized/ personal scale

⭘ Other

🞎 Criteria used in personal scale for OS:

⭘ Selection bias

⭘ Information Bias

⭘ Reporting Bias

⭘ Confounding Bias

⭘ Conflict of interest

⭘ Other

🞎 Was observational design considered by default at higher RoB compared to RCTs? ⭘ Yes

⭘ No

⭘ Unclear

***🡪 RCTs***

🞎 Tool’s for assessing RCTs:

⭘ Cochrane Risk of bias tool

⭘ Delphi List

⭘ PEDro scale

⭘ Jadad scale

⭘ Other/Personal scale

*3-Main results*

-Total number of studies included:

-Number of randomized controlled trials included:

-Number of all observational studies (OS) included, whatever the design:

-Which specific study design is included: 🞎 prospective study

🞎 retrospective study

🞎 case control study

🞎 case reports

🞎 interrupted time series studies`

🞎 cross-sectional study

🞎 electronic database

🞎 other

-Have they analyzed the different types of observational studies differently?

🞎 Yes

🞎 No

🞎 Unclear

***🡪 Risk Of Bias***

- Did they provide an evaluation of the risk of bias for primary studies included?

🞎 Yes for OS only

🞎 Yes for RCTs only

🞎 Yes for both

🞎 No

🞎 Unclear

-Were OS excluded from the SR if they are:

🞎 High ROB

🞎 Medium ROB

🞎 No exclusion

🞎 Unclear

***Methodological characteristics of Meta-analyses:*** 1 Pooled outcome

-number of RCTs used:

-number of OS used:

-What type of analysis did the outcome evaluate?  Efficacy

- - Safety

-What type of analysis did the outcome evaluate?  Primary

- - Secondary

-What type of data was pooled?  adjusted

- not adjusted
- unclear
- other
- not reported

-Which specific study design is included: 🞎 prospective study

🞎 retrospective study

🞎 case control study

🞎 case reports

🞎 interrupted time series studies`

🞎 cross-sectional study

🞎 electronic database

🞎 other

- Were OS excluded from the MA if they are:

🞎 High ROB

🞎 Medium ROB

🞎 No exclusion

🞎 Unclear

-did they provide estimate for each study design separately:

- Yes
- No
- If Yes: are the results consistent*?  Yes
  - No

*graphical evaluation: looking for similarities in the direction of the results

-was the weight of the studies reported?

- Yes for OS only
- Yes for RCTs only
- Yes for both
- No
- Unclear

-If yes, what was the weight of the studies?

- For OS
- For RCTs

## → Forest Plot

-Is the forest plot presented?

- Yes
- No
- did the forest plot contain the design type (RCT/OS)?
  - Yes
  - No
- Did the forest plot contain the effect of studies with precision?
  - Yes
  - No
- did the forest plot contain the weight of the studies?
  - Yes
  - No

## → Heterogeneity

-Did they find important heterogeneity for any of the outcomes:

- Yes
- No
- If Yes, how did they measure it?

-Did they consider the study design as a source of heterogeneity:  Yes

- - No

-Did they provide other potential explanations?

- Yes
- No
- If Yes, which?

*→* ***GRADE***

-Did they use GRADE?

- Yes, for RCT only
- Yes for OS only
- Yes for both

## Limitations

- No

-have they reported a limitation related to study design?

- - Yes
  - No

## → Abstract

-Are the results pooling RCT and OS presented in the abstract?

- - Yes
- No
- If Yes, was the effect number reported?  Yes
  - No

(1)

*→* ***Concerns***

-have they reported a concern in the synthesis of OS with RCT?

- Yes
- No
- If Yes, which ?
