# Supplementary material for: Synthesis methods used to combine observational studies and randomised trials in published meta-analyses
Source: Syst Rev. 2024 Feb 21;13:70. doi: 10.1186/s13643-024-02464-w (PMC10880204; doi:10.1186/s13643-024-02464-w)
Supplement: Supplementary file 3 — Additional file 3. References of 132 studies included. [file 13643_2024_2464_MOESM3_ESM.docx]

**Additional file 3**. References of 132 studies included

1. Adamson PB, Ginn G, Anker SD, Bourge RC, Abraham WT. Remote haemodynamic-guided care for patients with chronic heart failure: a meta-analysis of completed trials. Eur J Heart Fail. mars 2017;19(3):426‑33.

2. Afshin A, Peñalvo JL, Del Gobbo L, Silva J, Michaelson M, O’Flaherty M, et al. The prospective impact of food pricing on improving dietary consumption: A systematic review and meta-analysis. PLoS One. 2017;12(3):e0172277.

3. Almenawer SA, Badhiwala JH, Alhazzani W, Greenspoon J, Farrokhyar F, Yarascavitch B, et al. Biopsy versus partial versus gross total resection in older patients with high-grade glioma: a systematic review and meta-analysis. Neuro Oncol. juin 2015;17(6):868‑81.

4. An KR, Belley-Cote EP, Um KJ, Gupta S, McClure GR, Jaffer IH, et al. Antiplatelet Therapy versus Anticoagulation after Surgical Bioprosthetic Aortic Valve Replacement: A Systematic Review and Meta-Analysis. Thromb Haemost. févr 2019;119(2):328‑39.

5. Angeles CV, Kang R, Shirai K, Wong SL. Meta-analysis of completion lymph node dissection in sentinel lymph node-positive melanoma. Br J Surg. mai 2019;106(6):672‑81.

6. Arvaniti K, Lathyris D, Blot S, Apostolidou-Kiouti F, Koulenti D, Haidich AB. Cumulative Evidence of Randomized Controlled and Observational Studies on Catheter-Related Infection Risk of Central Venous Catheter Insertion Site in ICU Patients: A Pairwise and Network Meta-Analysis. Crit Care Med. avr 2017;45(4):e437‑48.

7. Badurdeen S, Marshall A, Daish H, Hatherill M, Berkley JA. Safety and Immunogenicity of Early Bacillus Calmette-Guérin Vaccination in Infants Who Are Preterm and/or Have Low Birth Weights: A Systematic Review and Meta-analysis. JAMA Pediatr. 1 janv 2019;173(1):75‑85.

8. Baker JJ, Öberg S, Andresen K, Klausen TW, Rosenberg J. Systematic review and network meta-analysis of methods of mesh fixation during laparoscopic ventral hernia repair. Br J Surg. janv 2018;105(1):37‑47.

9. Bashar K, Healy DA, Elsheikh S, Browne LD, Walsh MT, Clarke-Moloney M, et al. One-stage vs. two-stage brachio-basilic arteriovenous fistula for dialysis access: a systematic review and a meta-analysis. PLoS One. 2015;10(3):e0120154.

10. Bikdeli B, Chatterjee S, Desai NR, Kirtane AJ, Desai MM, Bracken MB, et al. Inferior Vena Cava Filters to Prevent Pulmonary Embolism: Systematic Review and Meta-Analysis. J Am Coll Cardiol. 26 sept 2017;70(13):1587‑97.

11. Billeter AT, Scheurlen KM, Probst P, Eichel S, Nickel F, Kopf S, et al. Meta-analysis of metabolic surgery versus medical treatment for microvascular complications in patients with type 2 diabetes mellitus. Br J Surg. févr 2018;105(3):168‑81.

12. Bonello L, Laine M, Lemesle G, Puymirat E, Dabry T, Thuny F, et al. Meta-Analysis of Potent P2Y12-ADP Receptor Antagonist Therapy Compared to Clopidogrel Therapy in Acute Coronary Syndrome Patients with Chronic Kidney Disease. Thromb Haemost. oct 2018;118(10):1839‑46.

13. Boulouis G, Labeyrie MA, Raymond J, Rodriguez-Régent C, Lukaszewicz AC, Bresson D, et al. Treatment of cerebral vasospasm following aneurysmal subarachnoid haemorrhage: a systematic review and meta-analysis. Eur Radiol. août 2017;27(8):3333‑42.

14. Buchanan PJ, Chieng LO, Hubbard ZS, Law TY, Chim H. Endoscopic versus Open In Situ Cubital Tunnel Release: A Systematic Review of the Literature and Meta-Analysis of 655 Patients. Plast Reconstr Surg. mars 2018;141(3):679‑84.

15. Chai-Adisaksopha C, Hillis C, Siegal DM, Movilla R, Heddle N, Iorio A, et al. Prothrombin complex concentrates versus fresh frozen plasma for warfarin reversal. A systematic review and meta-analysis. Thromb Haemost. 28 oct 2016;116(5):879‑90.

16. Chang SH, Kang YN, Chiu HY, Chiu YH. A Systematic Review and Meta-Analysis Comparing Pigtail Catheter and Chest Tube as the Initial Treatment for Pneumothorax. Chest. mai 2018;153(5):1201‑12.

17. Chen X, Chen K, He J, Yao K. Comparing the Curative Effects between Femtosecond Laser-Assisted Cataract Surgery and Conventional Phacoemulsification Surgery: A Meta-Analysis. PLoS One. 2016;11(3):e0152088.

18. Cheng C, Chen B, Xu H, Zhang Z, Xu W. Efficacy of concomitant acromioplasty in the treatment of rotator cuff tears: A systematic review and meta-analysis. PLoS One. 2018;13(11):e0207306.

19. Cheng F, Cen Y, Liu C, Liu R, Pan C, Dai S. Round versus Anatomical Implants in Primary Cosmetic Breast Augmentation: A Meta-Analysis and Systematic Review. Plast Reconstr Surg. mars 2019;143(3):711‑21.

20. Cheng X, Tian X, Yan Z, Jia M, Deng J, Wang Y, et al. Comparison of the Fertility Outcome of Salpingotomy and Salpingectomy in Women with Tubal Pregnancy: A Systematic Review and Meta-Analysis. PLoS One. 2016;11(3):e0152343.

21. Chirapongsathorn S, Valentin N, Alahdab F, Krittanawong C, Erwin PJ, Murad MH, et al. Nonselective β-Blockers and Survival in Patients With Cirrhosis and Ascites: A Systematic Review and Meta-analysis. Clin Gastroenterol Hepatol. août 2016;14(8):1096-1104.e9.

22. Couper K, Laloo R, Field R, Perkins GD, Thomas M, Yeung J. Prophylactic antibiotic use following cardiac arrest: A systematic review and meta-analysis. Resuscitation. août 2019;141:166‑73.

23. Couper K, Yeung J, Nicholson T, Quinn T, Lall R, Perkins GD. Mechanical chest compression devices at in-hospital cardiac arrest: A systematic review and meta-analysis. Resuscitation. juin 2016;103:24‑31.

24. D’Antonio F, Iacovelli A, Liberati M, Leombroni M, Murgano D, Cali G, et al. Role of interventional radiology in pregnancy complicated by placenta accreta spectrum disorder: systematic review and meta-analysis. Ultrasound Obstet Gynecol. juin 2019;53(6):743‑51.

25. de Milliano I, Twisk M, Ket JC, Huirne JA, Hehenkamp WJ. Pre-treatment with GnRHa or ulipristal acetate prior to laparoscopic and laparotomic myomectomy: A systematic review and meta-analysis. PLoS One. 2017;12(10):e0186158.

26. Devulapalli C, Jia Wei AT, DiBiagio JR, Baez ML, Baltodano PA, Seal SM, et al. Primary versus Flap Closure of Perineal Defects following Oncologic Resection: A Systematic Review and Meta-Analysis. Plast Reconstr Surg. mai 2016;137(5):1602‑13.

27. Diaz R, Hernandez-Vaquero D, Alvarez-Cabo R, Avanzas P, Silva J, Moris C, et al. Long-term outcomes of mechanical versus biological aortic valve prosthesis: Systematic review and meta-analysis. J Thorac Cardiovasc Surg. sept 2019;158(3):706-714.e18.

28. Felix CM, van den Berg VJ, Hoeks SE, Fam JM, Lenzen M, Boersma E, et al. Mid-term outcomes of the Absorb BVS versus second-generation DES: A systematic review and meta-analysis. PLoS One. 2018;13(5):e0197119.

29. Freedman SB, Xie J, Neufeld MS, Hamilton WL, Hartling L, Tarr PI, et al. Shiga Toxin-Producing Escherichia coli Infection, Antibiotics, and Risk of Developing Hemolytic Uremic Syndrome: A Meta-analysis. Clin Infect Dis. 15 mai 2016;62(10):1251‑8.

30. Gargiulo G, Sannino A, Capodanno D, Barbanti M, Buccheri S, Perrino C, et al. Transcatheter Aortic Valve Implantation Versus Surgical Aortic Valve Replacement: A Systematic Review and Meta-analysis. Ann Intern Med. 6 sept 2016;165(5):334‑44.

31. Gegia M, Winters N, Benedetti A, van Soolingen D, Menzies D. Treatment of isoniazid-resistant tuberculosis with first-line drugs: a systematic review and meta-analysis. Lancet Infect Dis. févr 2017;17(2):223‑34.

32. Gillis C, Buhler K, Bresee L, Carli F, Gramlich L, Culos-Reed N, et al. Effects of Nutritional Prehabilitation, With and Without Exercise, on Outcomes of Patients Who Undergo Colorectal Surgery: A Systematic Review and Meta-analysis. Gastroenterology. août 2018;155(2):391-410.e4.

33. Ha FJ, Nerlekar N, Cameron JD, Bennett MR, Meredith IT, West NEJ, et al. Midterm Safety and Efficacy of ABSORB Bioresorbable Vascular Scaffold Versus Everolimus-Eluting Metallic Stent: An Updated Meta-Analysis. JACC Cardiovasc Interv. 13 févr 2017;10(3):308‑10.

34. Hajibandeh S, Hajibandeh S, Antoniou SA, Torella F, Antoniou GA. Effect of beta-blockers on perioperative outcomes in vascular and endovascular surgery: a systematic review and meta-analysis. Br J Anaesth. janv 2017;118(1):11‑21.

35. Han MY, Yu AH, Yuan J, Cai XJ, Ren JB. Effect of anterior capsule polish on visual function: A meta-analysis. PLoS One. 2019;14(1):e0210205.

36. Harnoss JC, Zelienka I, Probst P, Grummich K, Müller-Lantzsch C, Harnoss JM, et al. Antibiotics Versus Surgical Therapy for Uncomplicated Appendicitis: Systematic Review and Meta-analysis of Controlled Trials (PROSPERO 2015: CRD42015016882). Ann Surg. mai 2017;265(5):889‑900.

37. Hilal T, Wang Z, Almader-Douglas D, Rosenthal A, Reeder CB, Jain T. Rituximab maintenance therapy for mantle cell lymphoma: A systematic review and meta-analysis. Am J Hematol. oct 2018;93(10):1220‑6.

38. Horita N, Otsuka T, Haranaga S, Namkoong H, Miki M, Miyashita N, et al. Beta-lactam plus macrolides or beta-lactam alone for community-acquired pneumonia: A systematic review and meta-analysis. Respirology. oct 2016;21(7):1193‑200.

39. Hsu YP, Hsu CW, Bai CH, Cheng SW, Chen KC, Chen C. Silodosin versus tamsulosin for medical expulsive treatment of ureteral stones: A systematic review and meta-analysis. PLoS One. 2018;13(8):e0203035.

40. Hu H, Qin B, He D, Lu Y, Zhao Z, Zhang J, et al. Regional versus General Anesthesia for Percutaneous Nephrolithotomy: A Meta-Analysis. PLoS One. 2015;10(5):e0126587.

41. Huang J, Yu Y, Wei C, Qin Q, Mo Q, Yang W. Harmonic Scalpel versus Electrocautery Dissection in Modified Radical Mastectomy for Breast Cancer: A Meta-Analysis. PLoS One. 2015;10(11):e0142271.

42. Huang J, Wang X, Chen X, Song Q, Liu W, Lu L. Perioperative Antibiotics to Prevent Acute Endophthalmitis after Ophthalmic Surgery: A Systematic Review and Meta-Analysis. PLoS One. 2016;11(11):e0166141.

43. Ide K, Yamada H, Kawasaki Y. Effect of gargling with tea and ingredients of tea on the prevention of influenza infection: a meta-analysis. BMC Public Health. 12 mai 2016;16:396.

44. Ierodiakonou D, Garcia-Larsen V, Logan A, Groome A, Cunha S, Chivinge J, et al. Timing of Allergenic Food Introduction to the Infant Diet and Risk of Allergic or Autoimmune Disease: A Systematic Review and Meta-analysis. JAMA. 20 sept 2016;316(11):1181‑92.

45. Ironside N, Barreto SG, Loveday B, Shrikhande SV, Windsor JA, Pandanaboyana S. Meta-analysis of an artery-first approach versus standard pancreatoduodenectomy on perioperative outcomes and survival. Br J Surg. mai 2018;105(6):628‑36.

46. Jesson J, Dahourou DL, Renaud F, Penazzato M, Leroy V. Adverse events associated with abacavir use in HIV-infected children and adolescents: a systematic review and meta-analysis. Lancet HIV. févr 2016;3(2):e64-75.

47. Kanters S, Socias ME, Paton NI, Vitoria M, Doherty M, Ayers D, et al. Comparative efficacy and safety of second-line antiretroviral therapy for treatment of HIV/AIDS: a systematic review and network meta-analysis. Lancet HIV. oct 2017;4(10):e433‑41.

48. Kim JS, Kwon SH. Mupirocin in the Treatment of Staphylococcal Infections in Chronic Rhinosinusitis: A Meta-Analysis. PLoS One. 2016;11(12):e0167369.

49. Komosa A, Lesiak M, Krasiński Z, Grygier M, Siniawski A, Skorupski W, et al. Optimal Timing of P2Y12 Inhibitor Loading in Patients Undergoing PCI: A Meta-Analysis. Thromb Haemost. juin 2019;119(6):1000‑20.

50. Kovacs SD, van Eijk AM, Sevene E, Dellicour S, Weiss NS, Emerson S, et al. The Safety of Artemisinin Derivatives for the Treatment of Malaria in the 2nd or 3rd Trimester of Pregnancy: A Systematic Review and Meta-Analysis. PLoS One. 2016;11(11):e0164963.

51. Kwak N, Park J, Kim E, Lee CH, Han SK, Yim JJ. Treatment Outcomes of Mycobacterium avium Complex Lung Disease: A Systematic Review and Meta-analysis. Clin Infect Dis. 1 oct 2017;65(7):1077‑84.

52. Lai WS, Rais-Bahrami S. Safety and Efficacy of En Bloc Renal Hilar Vascular Staple Ligation: A Meta-Analysis. J Urol. janv 2017;197(1):175‑81.

53. Lau A, Bradshaw CS, Lewis D, Fairley CK, Chen MY, Kong FYS, et al. The Efficacy of Azithromycin for the Treatment of Genital Mycoplasma genitalium: A Systematic Review and Meta-analysis. Clin Infect Dis. 1 nov 2015;61(9):1389‑99.

54. Li H, Liu C, Zhang H, Xu W, Liu J, Chen Y, et al. The Use of Unidirectional Barbed Suture for Urethrovesical Anastomosis during Robot-Assisted Radical Prostatectomy: A Systematic Review and Meta-Analysis of Efficacy and Safety. PLoS One. 2015;10(7):e0131167.

55. Li Z, Zhou Y, Xu Q, Chen X. Staged versus One-Time Complete Revascularization with Percutaneous Coronary Intervention in STEMI Patients with Multivessel Disease: A Systematic Review and Meta-Analysis. PLoS One. 2017;12(1):e0169406.

56. Lv Z, Li Y, Wu Y, Qu Y. Surgical complications of primary rhegmatogenous retinal detachment: a meta-analysis. PLoS One. 2015;10(3):e0116493.

57. Ma Q feng, Chu C biao, Song H qing. Intravenous versus intra-arterial thrombolysis in ischemic stroke: a systematic review and meta-analysis. PLoS One. 2015;10(1):e0116120.

58. Mainbourg S, Addario A, Samson M, Puéchal X, François M, Durupt S, et al. Prevalence of Giant Cell Arteritis Relapse in Patients Treated With Glucocorticoids: A Meta-Analysis. Arthritis Care Res (Hoboken). juin 2020;72(6):838‑49.

59. Malvankar-Mehta MS, Iordanous Y, Chen YN, Wang WW, Patel SS, Costella J, et al. iStent with Phacoemulsification versus Phacoemulsification Alone for Patients with Glaucoma and Cataract: A Meta-Analysis. PLoS One. 2015;10(7):e0131770.

60. Mariani J, Macchia A, De Abreu M, Gonzalez Villa Monte G, Tajer C. Multivessel versus Single Vessel Angioplasty in Non-ST Elevation Acute Coronary Syndromes: A Systematic Review and Metaanalysis. PLoS One. 2016;11(2):e0148756.

61. Matthews A, Stanway S, Farmer RE, Strongman H, Thomas S, Lyon AR, et al. Long term adjuvant endocrine therapy and risk of cardiovascular disease in female breast cancer survivors: systematic review. BMJ. 8 oct 2018;363:k3845.

62. Mattishent K, Thavarajah M, Sinha A, Peel A, Egger M, Solomkin J, et al. Safety of 80% vs 30-35% fraction of inspired oxygen in patients undergoing surgery: a systematic review and meta-analysis. Br J Anaesth. mars 2019;122(3):311‑24.

63. Mazine A, Friedrich JO, Nedadur R, Verma S, Ouzounian M, Jüni P, et al. Systematic review and meta-analysis of chordal replacement versus leaflet resection for posterior mitral leaflet prolapse. J Thorac Cardiovasc Surg. janv 2018;155(1):120-128.e10.

64. Mazine A, Rocha RV, El-Hamamsy I, Ouzounian M, Yanagawa B, Bhatt DL, et al. Ross Procedure vs Mechanical Aortic Valve Replacement in Adults: A Systematic Review and Meta-analysis. JAMA Cardiol. 1 oct 2018;3(10):978‑87.

65. Mesgarpour B, Heidinger BH, Roth D, Schmitz S, Walsh CD, Herkner H. Harms of off-label erythropoiesis-stimulating agents for critically ill people. Cochrane Database Syst Rev. 25 août 2017;8(8):CD010969.

66. Mongkhon P, Naser AY, Fanning L, Tse G, Lau WCY, Wong ICK, et al. Oral anticoagulants and risk of dementia: A systematic review and meta-analysis of observational studies and randomized controlled trials. Neurosci Biobehav Rev. janv 2019;96:1‑9.

67. Mu X, Wei J, A J, Li Z, Ou Y. The short-term efficacy and safety of artificial total disc replacement for selected patients with lumbar degenerative disc disease compared with anterior lumbar interbody fusion: A systematic review and meta-analysis. PLoS One. 2018;13(12):e0209660.

68. Müller-Stich BP, Kenngott HG, Gondan M, Stock C, Linke GR, Fritz F, et al. Use of Mesh in Laparoscopic Paraesophageal Hernia Repair: A Meta-Analysis and Risk-Benefit Analysis. PLoS One. 2015;10(10):e0139547.

69. Müller-Stich BP, Senft JD, Warschkow R, Kenngott HG, Billeter AT, Vit G, et al. Surgical versus medical treatment of type 2 diabetes mellitus in nonseverely obese patients: a systematic review and meta-analysis. Ann Surg. mars 2015;261(3):421‑9.

70. Murphy GRF, Gardiner MD, Glass GE, Kreis IA, Jain A, Hettiaratchy S. Meta-analysis of antibiotics for simple hand injuries requiring surgery. Br J Surg. avr 2016;103(5):487‑92.

71. Nagappa M, Mokhlesi B, Wong J, Wong DT, Kaw R, Chung F. The Effects of Continuous Positive Airway Pressure on Postoperative Outcomes in Obstructive Sleep Apnea Patients Undergoing Surgery: A Systematic Review and Meta-analysis. Anesth Analg. mai 2015;120(5):1013‑23.

72. Nair R, Perencevich EN, Blevins AE, Goto M, Nelson RE, Schweizer ML. Clinical Effectiveness of Mupirocin for Preventing Staphylococcus aureus Infections in Nonsurgical Settings: A Meta-analysis. Clin Infect Dis. 1 mars 2016;62(5):618‑30.

73. Noreikaite J, Jones P, Fitzpatrick J, Amitharaj R, Pietropaolo A, Vasdev N, et al. Fosfomycin vs. quinolone-based antibiotic prophylaxis for transrectal ultrasound-guided biopsy of the prostate: a systematic review and meta-analysis. Prostate Cancer Prostatic Dis. juin 2018;21(2):153‑60.

74. O’Donnell CM, McLoughlin L, Patterson CC, Clarke M, McCourt KC, McBrien ME, et al. Perioperative outcomes in the context of mode of anaesthesia for patients undergoing hip fracture surgery: systematic review and meta-analysis. Br J Anaesth. janv 2018;120(1):37‑50.

75. Ochen Y, Beks RB, van Heijl M, Hietbrink F, Leenen LPH, van der Velde D, et al. Operative treatment versus nonoperative treatment of Achilles tendon ruptures: systematic review and meta-analysis. BMJ. 7 janv 2019;364:k5120.

76. Özdemir-van Brunschot DMD, Koning GG, van Laarhoven KCJHM, Ergün M, van Horne SBCE, Rovers MM, et al. A comparison of technique modifications in laparoscopic donor nephrectomy: a systematic review and meta-analysis. PLoS One. 2015;10(3):e0121131.

77. Pan L, Chen M, Ji L, Zheng L, Yan P, Fang J, et al. The Safety and Efficacy of Laparoscopic Common Bile Duct Exploration Combined with Cholecystectomy for the Management of Cholecysto-choledocholithiasis: An Up-to-date Meta-analysis. Ann Surg. août 2018;268(2):247‑53.

78. Paul M, Dickstein Y, Raz-Pasteur A. Antibiotic de-escalation for bloodstream infections and pneumonia: systematic review and meta-analysis. Clin Microbiol Infect. déc 2016;22(12):960‑7.

79. Paul S, Saxena A, Terrin N, Viveiros K, Balk EM, Wong JB. Hepatitis B Virus Reactivation and Prophylaxis During Solid Tumor Chemotherapy: A Systematic Review and Meta-analysis. Ann Intern Med. 5 janv 2016;164(1):30‑40.

80. Perera M, Roberts MJ, Doi SAR, Bolton D. Prostatic urethral lift improves urinary symptoms and flow while preserving sexual function for men with benign prostatic hyperplasia: a systematic review and meta-analysis. Eur Urol. avr 2015;67(4):704‑13.

81. Peyrin-Biroulet L, Danese S, Argollo M, Pouillon L, Peppas S, Gonzalez-Lorenzo M, et al. Loss of Response to Vedolizumab and Ability of Dose Intensification to Restore Response in Patients With Crohn’s Disease or Ulcerative Colitis: A Systematic Review and Meta-analysis. Clin Gastroenterol Hepatol. avr 2019;17(5):838-846.e2.

82. Pierorazio PM, Johnson MH, Patel HD, Sozio SM, Sharma R, Iyoha E, et al. Management of Renal Masses and Localized Renal Cancer: Systematic Review and Meta-Analysis. J Urol. oct 2016;196(4):989‑99.

83. Price DR, Mikkelsen ME, Umscheid CA, Armstrong EJ. Neuromuscular Blocking Agents and Neuromuscular Dysfunction Acquired in Critical Illness: A Systematic Review and Meta-Analysis. Crit Care Med. nov 2016;44(11):2070‑8.

84. Qi J, Zhang P, Wang Y, Chen H, Li Y. Does Total Gastrectomy Provide Better Outcomes than Distal Subtotal Gastrectomy for Distal Gastric Cancer? A Systematic Review and Meta-Analysis. PLoS One. 2016;11(10):e0165179.

85. Qin M, Liu X, Wu SH, Zhang XD. Atrial Substrate Modification in Atrial Fibrillation: Targeting GP or CFAE? Evidence from Meta-Analysis of Clinical Trials. PLoS One. 2016;11(10):e0164989.

86. Qin X, Deng Y, Wu D, Yu L, Huang R. Does Enhanced External Counterpulsation (EECP) Significantly Affect Myocardial Perfusion?: A Systematic Review & Meta-Analysis. PLoS One. 2016;11(4):e0151822.

87. Rollins KE, Javanmard-Emamghissi H, Acheson AG, Lobo DN. The Role of Oral Antibiotic Preparation in Elective Colorectal Surgery: A Meta-analysis. Ann Surg. juill 2019;270(1):43‑58.

88. Roman M, Monaghan A, Serraino GF, Miller D, Pathak S, Lai F, et al. Meta-analysis of the influence of lifestyle changes for preoperative weight loss on surgical outcomes. Br J Surg. févr 2019;106(3):181‑9.

89. Schenone AL, Cohen A, Patarroyo G, Harper L, Wang X, Shishehbor MH, et al. Therapeutic hypothermia after cardiac arrest: A systematic review/meta-analysis exploring the impact of expanded criteria and targeted temperature. Resuscitation. nov 2016;108:102‑10.

90. Shen J, Huang KY, Zhu Y, Pan JW, Jiang H, Weng YX, et al. Effect of statin treatment on vasospasm-related morbidity and functional outcome in patients with aneurysmal subarachnoid hemorrhage: a systematic review and meta-analysis. J Neurosurg. août 2017;127(2):291‑301.

91. Sijbrandij M, Kleiboer A, Bisson JI, Barbui C, Cuijpers P. Pharmacological prevention of post-traumatic stress disorder and acute stress disorder: a systematic review and meta-analysis. Lancet Psychiatry. mai 2015;2(5):413‑21.

92. Smeeing DPJ, Houwert RM, Briet JP, Kelder JC, Segers MJM, Verleisdonk EJMM, et al. Weight-bearing and mobilization in the postoperative care of ankle fractures: a systematic review and meta-analysis of randomized controlled trials and cohort studies. PLoS One. 2015;10(2):e0118320.

93. Smith LM, Cozowicz C, Uda Y, Memtsoudis SG, Barrington MJ. Neuraxial and Combined Neuraxial/General Anesthesia Compared to General Anesthesia for Major Truncal and Lower Limb Surgery: A Systematic Review and Meta-analysis. Anesth Analg. déc 2017;125(6):1931‑45.

94. Spratt DE, Gordon Spratt EA, Wu S, DeRosa A, Lee NY, Lacouture ME, et al. Efficacy of skin-directed therapy for cutaneous metastases from advanced cancer: a meta-analysis. J Clin Oncol. 1 oct 2014;32(28):3144‑55.

95. Stahl M, Bewersdorf JP, Giri S, Wang R, Zeidan AM. Use of immunosuppressive therapy for management of myelodysplastic syndromes: a systematic review and meta-analysis. Haematologica. janv 2020;105(1):102‑11.

96. Stecher M, Claßen A, Klein F, Lehmann C, Gruell H, Platten M, et al. Systematic Review and Meta-analysis of Treatment Interruptions in Human Immunodeficiency Virus (HIV) Type 1-infected Patients Receiving Antiretroviral Therapy: Implications for Future HIV Cure Trials. Clin Infect Dis. 17 mars 2020;70(7):1406‑17.

97. Steinemann DC, Müller PC, Probst P, Schwarz AC, Büchler MW, Müller-Stich BP, et al. Meta-analysis of hybrid natural-orifice transluminal endoscopic surgery versus laparoscopic surgery. Br J Surg. juill 2017;104(8):977‑89.

98. Stephens RJ, Dettmer MR, Roberts BW, Ablordeppey E, Fowler SA, Kollef MH, et al. Practice Patterns and Outcomes Associated With Early Sedation Depth in Mechanically Ventilated Patients: A Systematic Review and Meta-Analysis. Crit Care Med. mars 2018;46(3):471‑9.

99. Suthar AB, Vitoria MA, Nagata JM, Anglaret X, Mbori-Ngacha D, Sued O, et al. Co-trimoxazole prophylaxis in adults, including pregnant women, with HIV: a systematic review and meta-analysis. Lancet HIV. avr 2015;2(4):e137-150.

100. Syn NL, Wee I, Shabbir A, Kim G, So JBY. Pouch Versus No Pouch Following Total Gastrectomy: Meta-analysis of Randomized and Non-randomized Studies. Ann Surg. juin 2019;269(6):1041‑53.

101. Te Morenga L, Montez JM. Health effects of saturated and trans-fatty acid intake in children and adolescents: Systematic review and meta-analysis. PLoS One. 2017;12(11):e0186672.

102. Teerawattanapong N, Kengkla K, Dilokthornsakul P, Saokaew S, Apisarnthanarak A, Chaiyakunapruk N. Prevention and Control of Multidrug-Resistant Gram-Negative Bacteria in Adult Intensive Care Units: A Systematic Review and Network Meta-analysis. Clin Infect Dis. 15 mai 2017;64(suppl_2):S51‑60.

103. Thorlund JB, Juhl CB, Roos EM, Lohmander LS. Arthroscopic surgery for degenerative knee: systematic review and meta-analysis of benefits and harms. BMJ. 16 juin 2015;350:h2747.

104. Tian DH, Weller J, Hasmat S, Oo A, Forrest P, Kiat H, et al. Adjunct retrograde cerebral perfusion provides superior outcomes compared with hypothermic circulatory arrest alone: A meta-analysis. J Thorac Cardiovasc Surg. oct 2018;156(4):1339-1348.e7.

105. Toyota T, Morimoto T, Shiomi H, Yoshikawa Y, Yaku H, Yamashita Y, et al. Very Late Scaffold Thrombosis of Bioresorbable Vascular Scaffold: Systematic Review and a Meta-Analysis. JACC Cardiovasc Interv. 9 janv 2017;10(1):27‑37.

106. Traeger MW, Schroeder SE, Wright EJ, Hellard ME, Cornelisse VJ, Doyle JS, et al. Effects of Pre-exposure Prophylaxis for the Prevention of Human Immunodeficiency Virus Infection on Sexual Risk Behavior in Men Who Have Sex With Men: A Systematic Review and Meta-analysis. Clin Infect Dis. 16 août 2018;67(5):676‑86.

107. Tran-Duy A, Spaetgens B, Hoes AW, de Wit NJ, Stehouwer CDA. Use of Proton Pump Inhibitors and Risks of Fundic Gland Polyps and Gastric Cancer: Systematic Review and Meta-analysis. Clin Gastroenterol Hepatol. déc 2016;14(12):1706-1719.e5.

108. Trastulli S, Cirocchi R, Desiderio J, Coratti A, Guarino S, Renzi C, et al. Robotic versus Laparoscopic Approach in Colonic Resections for Cancer and Benign Diseases: Systematic Review and Meta-Analysis. PLoS One. 2015;10(7):e0134062.

109. Veroniki AA, Cogo E, Rios P, Straus SE, Finkelstein Y, Kealey R, et al. Comparative safety of anti-epileptic drugs during pregnancy: a systematic review and network meta-analysis of congenital malformations and prenatal outcomes. BMC Med. 5 mai 2017;15(1):95.

110. Virk SA, Tian DH, Sriravindrarajah A, Dunn D, Wolfenden HD, Suri RM, et al. Mitral valve surgery and coronary artery bypass grafting for moderate-to-severe ischemic mitral regurgitation: Meta-analysis of clinical and echocardiographic outcomes. J Thorac Cardiovasc Surg. juill 2017;154(1):127‑36.

111. Visioni A, Shah R, Gabriel E, Attwood K, Kukar M, Nurkin S. Enhanced Recovery After Surgery for Noncolorectal Surgery?: A Systematic Review and Meta-analysis of Major Abdominal Surgery. Ann Surg. janv 2018;267(1):57‑65.

112. Volovici V, Huijben JA, Ercole A, Stocchetti N, Dirven CMF, van der Jagt M, et al. Ventricular Drainage Catheters versus Intracranial Parenchymal Catheters for Intracranial Pressure Monitoring-Based Management of Traumatic Brain Injury: A Systematic Review and Meta-Analysis. J Neurotrauma. 1 avr 2019;36(7):988‑95.

113. Wang Q, Guo J, Hu H, Lu Y, Zhang J, Qin B, et al. Rigid ureteroscopic lithotripsy versus percutaneous nephrolithotomy for large proximal ureteral stones: A meta-analysis. PLoS One. 2017;12(2):e0171478.

114. Weibel S, Neubert K, Jelting Y, Meissner W, Wöckel A, Roewer N, et al. Incidence and severity of chronic pain after caesarean section: A systematic review with meta-analysis. Eur J Anaesthesiol. nov 2016;33(11):853‑65.

115. Wong B, Muneer M, Wiebe N, Storie D, Shurraw S, Pannu N, et al. Buttonhole versus rope-ladder cannulation of arteriovenous fistulas for hemodialysis: a systematic review. Am J Kidney Dis. déc 2014;64(6):918‑36.

116. Wu Z, Zhang H, Jin W, Liu Y, Lu L, Chen Q, et al. The Effect of Renin-Angiotensin-Aldosterone System Blockade Medications on Contrast-Induced Nephropathy in Patients Undergoing Coronary Angiography: A Meta-Analysis. PLoS One. 2015;10(6):e0129747.

117. Xu K, Chan NC, Ibrahim Q, Kruger P, Sinha S, Bhagirath V, et al. Reduction in Mortality following Elective Major Hip and Knee Surgery: A Systematic Review and Meta-Analysis. Thromb Haemost. avr 2019;119(4):668‑74.

118. Xu Z, Cao D, Chen X, Wu S, Wang X, Wu Q. Comparison of clinical performance between trifocal and bifocal intraocular lenses: A meta-analysis. PLoS One. 2017;12(10):e0186522.

119. Yamada T, Okabayashi K, Hasegawa H, Tsuruta M, Yoo JH, Seishima R, et al. Meta-analysis of the risk of small bowel obstruction following open or laparoscopic colorectal surgery. Br J Surg. avr 2016;103(5):493‑503.

120. Yang H, Zhang C, Zhou Q, Wang Y, Chen L. Clinical outcomes with alternative dosing strategies for piperacillin/tazobactam: a systematic review and meta-analysis. PLoS One. 2015;10(1):e0116769.

121. Yang R, Zhao X, Yang Y, Huang X, Li H, Su L. The efficacy and safety of pharmacologic thromboprophylaxis following caesarean section: A systematic review and meta-analysis. PLoS One. 2018;13(12):e0208725.

122. Yu Z, Pang X, Wu X, Shan C, Jiang S. Clinical outcomes of prolonged infusion (extended infusion or continuous infusion) versus intermittent bolus of meropenem in severe infection: A meta-analysis. PLoS One. 2018;13(7):e0201667.

123. Yuan Q, Wu X, Sun Y, Yu J, Li Z, Du Z, et al. Impact of intracranial pressure monitoring on mortality in patients with traumatic brain injury: a systematic review and meta-analysis. J Neurosurg. mars 2015;122(3):574‑87.

124. Zhang XL, Zhu L, Wei ZH, Zhu QQ, Qiao JZ, Dai Q, et al. Comparative Efficacy and Safety of Everolimus-Eluting Bioresorbable Scaffold Versus Everolimus-Eluting Metallic Stents: A Systematic Review and Meta-analysis. Ann Intern Med. 7 juin 2016;164(11):752‑63.

125. Zhang XL, Zhu QQ, Kang LN, Li XL, Xu B. Mid- and Long-Term Outcome Comparisons of Everolimus-Eluting Bioresorbable Scaffolds Versus Everolimus-Eluting Metallic Stents: A Systematic Review and Meta-analysis. Ann Intern Med. 7 nov 2017;167(9):642‑54.

126. Zhang XL, Zhu QQ, Yang JJ, Chen YH, Li Y, Zhu SH, et al. Percutaneous intervention versus coronary artery bypass graft surgery in left main coronary artery stenosis: a systematic review and meta-analysis. BMC Med. 21 avr 2017;15(1):84.

127. Zhao SJ, Zhong ZS, Qi GX, Shi LY, Chen L, Tian W. Effect of Pioglitazone in Preventing In-Stent Restenosis after Percutaneous Coronary Intervention in Patients with Type 2 Diabetes: A Meta-Analysis. PLoS One. 2016;11(5):e0155273.

128. Zheng G, Li S, Huang M, Liu F, Tao J, Chen L. The effect of Tai Chi training on cardiorespiratory fitness in healthy adults: a systematic review and meta-analysis. PLoS One. 2015;10(2):e0117360.

129. Zhou C, Zhang L, Wang H, Ma X, Shi B, Chen W, et al. Superiority of Minimally Invasive Oesophagectomy in Reducing In-Hospital Mortality of Patients with Resectable Oesophageal Cancer: A Meta-Analysis. PLoS One. 2015;10(7):e0132889.

130. Zhou P, Zhu P, Nie Z, Zheng S. Is the era of bilateral internal thoracic artery grafting coming for diabetic patients? An updated meta-analysis. J Thorac Cardiovasc Surg. déc 2019;158(6):1559-1570.e2.

131. Zhu XC, Yu Y, Wang HF, Jiang T, Cao L, Wang C, et al. Physiotherapy intervention in Alzheimer’s disease: systematic review and meta-analysis. J Alzheimers Dis. 2015;44(1):163‑74.

132. Ziff OJ, Lane DA, Samra M, Griffith M, Kirchhof P, Lip GYH, et al. Safety and efficacy of digoxin: systematic review and meta-analysis of observational and controlled trial data. BMJ. 30 août 2015;351:h4451.
